# Inversion of the Uterus Following Parturition

**Published:** 1856-05

**Authors:** 


We find in the American Journal of Medical Sciences, for April, a series of cases of Inversion of the Uterus, which we introduce as illustrating the position we have taken in answer to our correspondent, Dr. Jamieson. We italicise portions.

*Inversion of the Uterus following Parturition.*—Two cases of this rare and alarming accident, are recorded in the Association Medical Journal, during the past year, one by Mr. W. B. Borham, and the second by Mr. C. Leonard.

*Mr. Borham's Case.* Mrs. H., aged 25, a fine strong woman, was delivered of her first child with the *funis twice twisted* round the child's neck, on January 31st, at 3, P. M., by Mrs. Collen, of Paddington street, a midwife of considerable experience, after a natural labor of sixteen hours' duration. The pains occurred at rather longer intervals than usual, and were very forcing. About an hour after the child was born, I arrived in consequence of a hasty summons, when the patient presented the following state: The whole of the uterus, dragging a small portion of the posterior part of the vagina with it, was lying (like a scrotal hernia) without the os externum; it was covered with the deciduary and other membranes, and the placenta was entirely attached to its fundus. The patient was lying upon her back, with her knees bent up. She was pulseless, had difficulty of breathing, was cold, prostrated, and exhausted; in fact, in a state of collapse. The uterus had been exposed for three-quarters of an hour, and had become much contracted. *I immediately detached the placenta by inserting the index finger of my right hand between it and the uterus*, and then peeled off the membranes attached to it (those which spring from the placenta.) The midwife now well greased my left arm and hand, with which I grasped the uterus, and returned it within the vagina. I then withdrew my hand a little, *formed my fingers into a cone, reintroduced it, gently carrying the fundus before me until I had carried it up to a level with the patient's umbilicus*, when I had the satisfaction of finding that the uterus had righted itself into its normal position in the pelvis. Its neck and os then commenced a gradual contraction on the fingers, when I carefully and steadily withdrew my hand, desiring the midwife at the same time to make pressure just above the pubes with her hand. After the withdrawal of mine, I had a saucer placed upon the lower part of the abdomen, and a bandage bound tightly over it.

During this time, which lasted about fifteen minutes, the patient was in a state of great exhaustion, and it was only by the aid of brandy that I could at all arouse her. I remained with her an hour, by which time she rallied considerably. I then left, prescribing for her a mixture of sulphuric acid, sulphuric ether, and camphor mixture, every two hours.

At ten o'clock I was again sent for, as a return was feared; but, on examining, I found it was only escaping, and I placed a sponge in the vagina.

Feb. 1st. The patient remained in a very low state; the pulse was 150, and feeble; she was very thirsty; the discharge was moderate. She was ordered to continue the mixture.

2d. The pulse was 120. The patient was very excited, with peritonitis threatening. I ordered two grains of opium and five of calomel to be taken directly, and a saline mixture with prussic acid. The patient had a desire to micturate, and for the first time I desired her to be moved for that purpose, when she passed a great quantity of urine. She felt exhausted, but much relieved.

3d. The pulse was 110. She had had four very liquid stools in the night which produced faintness. She complained of much pain in the bowels, and was restless. The tongue was furred; there was a dragging or tearing pain in the bowels; the discharge was offensive and purulent. I ordered fifteen leeches to be applied to the epigastrium, and a poultice afterward; I also prescribed gray and Dover's powders to be taken every three hours, and an effervescing saline draught with an excess of ammonia and prussic acid every four hours.

4th. She was better in every respect. The medicines were continued.

5th. She was progressing favorably.

She continued daily to get better. Her milk came at the ninth day; and I left her convalescent at the end of three weeks.

*Remarks.* The midwife assured me that she did not use the slightest traction at the funis after the birth of the child, but that the uterus and attached membranes came out all at once with one pain about half an hour after the child was expelled; and she, thinking it a "tumor," desired I might be instantly sent for. The funis being twice convoluted round the child's neck, greatly reduced its remaining length, and doubtless this circumstance assisted considerably in dragging down the fundus, particularly as the pains were described as "very forcing;" the fundus thus becoming cup-shaped from unavoidable traction, the whole uterus soon became inverted and expelled.

There is a division of opinion as to the advisability of detaching the placenta from an inverted womb before returning it Much must of course depend upon the contracted state of this organ; the length of time it has been exposed, etc.; but I think the placenta should always be separated before returning the uterus, if the latter be in a *contracted state;* for the less the substance to be returned the easier is it accomplished. Nor need we fear hæmorrhage, for the uterus being inverted would drag down the uterine arteries into its inverted cavity; they would become intussuscepted, the uterus contracting upon them, forming an artificial tourniquet; and thus the supply to the uterine sinuses would be cut off (as was the case in this instance, for there was no hæmorrhage during the operation of detachment.) I therefore cannot think, with some authorities, that the patient is sure speedily to die of profuse hæmorrhage if the placenta be detached. On the other hand, if the uterus were in a very relaxed state, and its os well dilated or easily dilatable, the hæmorrhage might indeed be great if the placenta were partly detached; and in such a state there would be little difficulty in returning the whole mass, and, perhaps, it would be advisable to do so. But in a case like my own it was desirable to return the uterus with as little addition to it as possible, owing to the contracted state of that tissue.

That the uterus should be *immediately* returned there cannot be two opinions; or its contraction would soon prevent its return. The diminished size of the os would prevent the fundus from returning through it to its normal position, and strangulation would ensue, with its evil consequences. That peritonitis ensued in my case is not to be wondered at, considering how the peritoneum must have been interfered with by such a sudden revulsion of its tissue. The favorable termination of the case will certainly add a good practical confirmation to the opinion of those who are in favor of removing the placenta before returning the uterus. The uterus did not return with a sudden jerk, such as is usually ascribed to such cases.

*Mr. Leonard's Case.* The subject of this case was a lady, aged 34 years, a stout, healthy person, who had previously had one child, still-born, after a tedious labor, from abscence of pains, requiring the application of the forceps.

April 25, 1855. For the last two days there has been some slight watery discharge, but no pains till 2, P. M., to-day. Her ordinary medical attendant was sent for at 4, P. M., who found the membranes ruptured, the os uteri fully dilated, and the head descending, the presentation being natural. The pains continued strong till the child was born, about 8½, P. M. The child, a female, was very large, and living; no hæmorrhage followed its expulsion. The funis was very short, twice round the neck, and so tightly strained, that it was impossible to relieve it, or even apply ligatures, the child being so closely confined to the body of the mother; it was, therefore, divided, when a portion was immediately retracted within the vagina. No pains followed; and, on examination in about twenty minutes, the insertion of the cord was felt very low down, and a strong expulsive effort was followed by the protrusion of a large mass, which proved to be the uterus inverted, with the placenta completely attached; this mass gradually increased in size, from hæmorrhage occurring between the centre of the placenta and the uterus, the edges of the former remaining adherent. Whilst supporting this mass, it suddenly gave way about the centre, giving exit to a quantity of coagula and fluid blood. The patient now became very faint and exhausted.

At this time I saw her. On introducing the finger within the vagina, a hard mass was felt, which the finger could freely pass round, and which was the inverted vagina. *I immediately detached the placenta, and the hæmorrhage ceased,* the uterus, which lay completely exposed, looking pale. I I then returned it within the vagina, and introducing my right hand, *grasped the fundus uteri, and gradually pushed it up within the os,* restoring it to its normal position.

I kept my hand firmly pressed over the uterus for some time, and very little hæmorrhage occurred. A dose of ergot was administered, and brandy and gruel given freely, as she continued faint for some time. She subsequently made a good recovery, sitting up for the first time on May 19th.

This was clearly a case of unavoidable inversion, induced by the traction exerted upon the uterus by an originally short funis, further shortened by the circumstances before mentioned, and which no interference could have prevented.

*Abortion: Spontaneous Inversion of the Uterus.*—Dr. John A. Brady reports (*New York Medical Times,* February, 1856,) a case of this. The subject of it was an unmarried woman, aged 23, who had had a child two years before, and five months advanced in her second pregnancy. When seen by Dr. B., the patient had powerful labor pains, the os was fully dilated, the membranes protruding, and the head presenting. She stated that three days previously, while walking with a friend, she made a mis-step, it being very dark, falling on her abdomen and bruising it severely; and that about twenty-four hours before Dr. B.'s visit, she was severely kicked on the abdomen by her brother, who was enraged at finding her in a pregnant condition: she had been in pain ever since the receipt of the last blow, but did not think she was in labor until about five hours before Dr. B. visited her. The pains continued in frequency and force until 4½, A. M., when the membranes ruptured, and at 6, A. M., after a very severe pain, she was delivered of a five months female fœtus, which lived but a moment. The cord was of the usual length, and not wound around the neck of the child. Just after the cord was severed, and while Dr. B. was removing the child from the bed, she was seized with alarming and frightful hæmorrhage. The bed and its clothing were saturated, and even the floor under the bed covered by the vital fluid; the case appeared to be hopeless; the pulse was not perceptible at the wrist, the face was blanched and death-like, the features pinched, the breathing laborious, the body cold and bathed in perspiration, and the woman apparently dying. The uterus was found lying between the thighs, inverted, with the placenta attached to its fundus; it was flaccid, and not in a contracted condition. Dr. B. immediately restored it to the vagina, *and by forming his fingers into a cone and pressing them against the fundus, succeeded with very little difficulty in restoring the organ*, with the placenta still attached, to its proper position. Dr. B. kept his hand in the uterus until the patient had rallied somewhat, and contraction of the organ had taken place. Before removing it, he tried with his fingers to peel off the placenta, but being firmly adherent, he only succeeded in getting away about one-half of the central portion of it; and being fearful of causing a return of the flooding if much force was used—knowing that a further loss of blood, however slight, would cause his patient to sink and die, he desisted from the attempt, and allowed it to remain. During all this time, brandy, and subsequently carb. ammon. were used freely, together with the external application of dry heat and the use of friction. A bandage was also carefully applied to the abdomen, and the woman kept perfectly still and quiet on her back, with the hips elevated. The hæmorrhage at this stage was trifling; reäction came on gradually, and at noon her cheeks were hot; pulse 140, full and sharp, and there was some delirium present. The uterus was firmly contracted on the placenta, which thus far had resisted every attempt made for its removal. At 3, P. M., the patient being placed under the influence of chloroform, Dr. B. satisfied himself by a careful examination of the impossibility of removing the placenta without resorting to greater violence than would have been justifiable, he determined to let it remain where it was, and wait for its removal, —in the meanwhile feeding her on brandy and beef tea, and using frequent vaginal injections.

Sept. 19, afternoon. Patient began to have considerable pain in back and abdomen, accompanied by a florid discharge of blood from the uterus. Administered ʒj. of tinct. ergot. sat.; and after oiling his hand well and passing it up the vagina, Dr. B. succeeded, with a great deal of difficulty, in bringing away the remainder of the placenta; it was in a putrid condition, and emitted a horrible odor. The hæmorrhage was slight, and caused no marked prostration; an injection of cold water was then carefully thrown up the vagina, the bandage adjusted, and the patient kept quiet in the recumbent posture. From this time she slowly recovered.

We consider the return of the placenta with the uterus an error in practice. In this case it was *once* capable of being easily removed, but Dr. B., in following the rule of authors in this case, involved the patient in serious trouble.

